# Knowledge and emotional attitudes of health care practitioners regarding patients with psychogenic nonepileptic seizures

**DOI:** 10.1055/s-0042-1758646

**Published:** 2022-12-28

**Authors:** Thani Sheikh Saker, Mark Katson, Sari Eran Herskovitz, Moshe Herskovitz

**Affiliations:** 1 Rambam Health Care Center, Neurology Department, Haifa, Israel.; 2 Ministry of Education, Department of Special Education, Afula, Israel.; 3 Technion Institute of Technology, Faculty of Medicine, Haifa, Israel.

**Keywords:** Attitude, Seizures, Health Personnel, Health Surveys, Atitude, Convulsões, Pessoal de Saúde, Inquéritos Epidemiológicos

## Abstract

**Background** Psychogenic nonepileptic seizures (PNESs) are paroxysmal events that resemble epileptic seizures without concomitant changes in electroencephalograms (EEGs) or any other physiological cause. These seizures are one of the most common and dramatic conversion disorders. First responders treat many PNES patients with unnecessary emergency abortive medication and sometimes even intubate them. Several of our PNES patients have complained they have experienced harsh attitudes from health care practitioners (HCPs), especially during their stay in the emergency room (ER).

**Objective** To assess the emotional attitude of HCPs toward PNES patients.

**Methods** We handed a questionnaire containing 23 questions regarding PNES patients to HCPs from emergency medicine, internal medicine, and neurology disciplines. The questions dealt with three categories: diagnosis, management, and emotional attitudes.

**Results** Overall, 47 HCPs participated in this study: 11 ER, 18 internal medicine, and 18 neurology practitioners. The HCP from those disciplines showed high knowledge and good management practice of PNES patients. On the other hand, the HCPs agreed with most emotional attitude statements regarding PNES patients, reflecting a high percentage of negative emotional attitudes toward this group of patients. We did not find any correlation between negative emotional attitude and HCP department, profession, or seniority.

**Conclusions** Although HCPs in our center perform good management practice regarding PNES patients, most reported a negative emotional attitude. This finding implies that what PNES patients feel regarding the harsh attitudes is also reflected by HCP views. Emotional attitudes towards PNES patients may not depend solely on the level of knowledge of the HCPs.

## Resumo

**Antecedentes** Crises não epilépticas psicogênicas (CNEPs) são eventos paroxísticos que se assemelham a crises epilépticas sem alterações concomitantes nos eletroencefalogramas (EEGs) ou não causadas por qualquer outra causa fisiológica. Essas convulsões são um dos distúrbios de conversão mais comuns e dramáticos. Os socorristas tratam muitos pacientes com CNEP com medicamentos abortivos de emergência desnecessariamente e às vezes até os entubam. Vários de nossos pacientes com CNEP queixaram-se de atitudes duras dos profissionais de saúde (HCPs), especialmente durante a permanência na sala de emergência (SE).

**Objetivo** Avaliar a atitude emocional dos profissionais de saúde em relação aos pacientes com CNEP.

**Métodos** Entregamos um questionário contendo 23 perguntas sobre pacientes com CNEP aos profissionais de saúde das disciplinas de medicina de emergência, medicina interna e neurologia. As questões tratavam de três categorias: diagnóstico, manejo e atitudes emocionais.

**Resultados** No geral, 47 profissionais de saúde participaram deste estudo: 11 ER, 18 medicina interna e 18 profissionais de neurologia. Os profissionais de saúde dessas disciplinas apresentaram alto conhecimento e boa prática de manejo dos pacientes da CNEP. Por outro lado, os profissionais de saúde concordaram com as afirmações da maioria das atitudes emocionais em relação aos pacientes da CNEP, refletindo um alto percentual de atitudes emocionais negativas em relação a esse grupo de pacientes. Não encontramos nenhuma correlação entre atitude emocional negativa e departamento de HCP, profissão ou antiguidade.

**Conclusões** Embora os profissionais de saúde de nosso centro tenham uma boa prática profissional de manejo em relação aos pacientes com CNEP, a maioria relatou uma atitude emocional negativa. Esse achado implica que o que os pacientes da PNES sentem em relação às atitudes duras também se reflete na visão do HCP. Atitudes emocionais em relação aos pacientes da CNEP podem não depender apenas do nível de conhecimento dos profissionais de saúde.

## INTRODUCTION

Psychogenic nonepileptic seizures (PNESs) are paroxysmal events that clinically resemble epileptic seizures without the concomitant changes in electroencephalograms (EEGs) or any other physiological cause. 1

Due to its similarity to epileptic seizures (ESs), PNES is difficult to diagnose. The mean latency from onset to correct diagnosis is of up to 7.5 years. 2

Health care practitioners (HCPs) from various disciplines, including internal medicine, emergency medicine, and neurology encounter PNES patients in their clinical routine.

These seizures are one of the most common and dramatic conversion/somatoform disorders; first responders treat many PNES patients with unnecessary emergency abortive medication and sometimes even intubate them. 3 As a tertiary epilepsy center, we encounter many patients with PNES, 4 and several of them have complained that they have experienced a harsh attitude from HCPs, especially during their stay in the Emergency Room (ER.)

In our previous study, 5 we demonstrated that ER personnel have difficulty differentiating between ES and PNES. We concluded that there is a need for better education of first responders and to record the episodes on video. Other studies 6 7 8 9 10 have reached the same conclusion.

Motivated by the subjective feelings of our PNES patients, we conducted the present study to check the emotional attitude of HCPs toward PNES patients.

## METHODS

The present is a single-center study. We handed a questionnaire containing 23 questions ( Table 1 ) to nurses and doctors from internal medicine, emergency medicine, and the neurology department. The participants filled out the questionnaire in their spare time and returned it by hand.

**Table 1:** Questionnaire applied in the present study

Number	Question/statement
1	The term pseudo-seizure is appropriate to use to describe psychogenic non-epileptic seizures (PNESs).
2	Medical staff can comfortably diagnose PNES based on clinical history.
3	Medical staff can differentiate PNES from epileptic seizures once witnessing the event.
4	Diagnosis of PNES must always be confirmed by long term video-electroencephalographic monitoring.
5	Inducing the patient's events at bedside (by suggestion or doing certain maneuvers) confirms that the events are PNES.
6	If I can, I try to induce an event.
7	If prolactin or creatine kinase (CK) level is not elevated in a patient with a spell, this confirms PNES.
8	Patients with PNES have control of their seizures.
9	Patients with PNES can also have epileptic seizures.
10	PNES occurs more commonly in women.
11	PNES occurs more commonly in minorities.
12	PNES patients require attention beyond what is justified by their medical condition.
13	PNES patients misuse medical resources.
14	PNES patients waste the time of the medical staff.
15	PNES patients often arouse anger among the medical staff.
16	PNES patients are treated disrespectfully by the medical staff.
17	PNES patients are less urgent.
18	Suspected PNES patients should first be referred to a neurologist.
19	Suspected PNES patients should first be referred to a psychiatrist or psychologist.
20	ASM is the best treatment for PNES patients
21	Patients with PNES must have their driving privileges restricted like patients with epileptic seizures
22	Anti- seizure medications (ASMs) should be discontinued in patients with PNES.
23	It is difficult for PNES patients to accept the diagnosis.

We included HCPs from those disciplines since they serve as first responders in cases of patients with suspected seizures.

We based the questionnaire on previously reported surveys. 8 9 10 11

We used a Likert scale, and each question was graded from 1 to 5: 1–strongly disagree; 2–disagree; 3–neither agree nor disagree; 4–agree; and 5–strongly agree. To maintain uniformity, we used the same scale in all questions in the questionnaire itself. During the analysis, questions 1 to 11 and 18 to 23 were scaled down to disagree (1-2), neutral (3), and agree (4-5).

- Questions 1-11 dealt with HCP's diagnosis making and knowledge regarding PNES patients. Questions 18-23 dealt with HCP's management of PNES patients. Both groups of questions served to map the first responders' knowledge regarding PNES patients and understand how they manage PNES patients at our center. We believe that this mapping could help to improve the quality of the care provided to these patients.
- Questions 12-17 dealt with HCP's emotional attitudes towards PNES patients. This section served as the primary goal of our survey and checked whether the views of the HCPs reflect our patients' subjective feelings. The questions in this section were asked in the third person to ensure the full cooperation of responders. In each question, a score above 3 implied a negative emotional attitude.

For the descriptive and inferential statistics, we used the Statistical Package for the Social Sciences (IBM SPSS Statistics for Windows, IBM Corp., Armonk, NY, United States), software, version 25.0.

We performed univariate analysis to test the differences and interactions regarding means. We used the Pearson correlation test to check the linear correlation.

Our Institutional Review Board approved all study procedures.

## RESULTS

Overall, we handed 65 questionnaires, which were handed back by 47 (72%) participants, who included: 11 ER professionals, 18 internal medicine professionals, and 18 neurology professionals. There were 18 nurses and 29 physicians, 23 male and 24 female participants. The seniority of the participants varied from less than 1 year to 30 years, with mean of 8.8 ± 9.8 years. Table 2 summarizes the participant's demographic data.

**Table 2:** Demographics of the study sample

Variable	Values	Frequency (n)	Valid percentage (%)	Mean	Standard deviation
Profession	Nurse	18	38.3		
	Doctor of Medicine	29	61.7		
Gender	Male	23	48.9		
	Female	24	51.1		
Department	Neurology	18			
	Emergency Room	11			
	Internal Wards	18			
Seniority (years)				8.8	9.8

**Figure 1:**
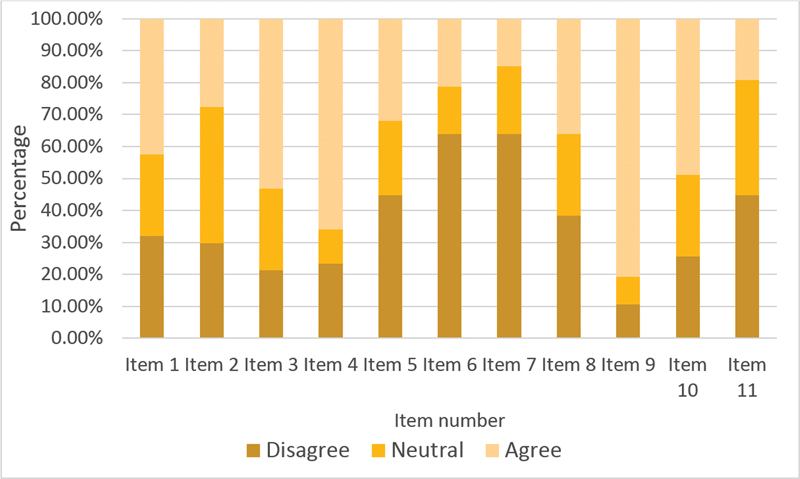
Percentage of agreement with items 1 to 11.

Figure 1 summarizes the results of items regarding knowledge and diagnosis making in patients with PNES. Question 9 was the item most agreed (81%) upon: “Patients with PNES can also have epileptic seizures”. Question 7 was the item most disagreed upon (63%): “If prolactin or creatine kinase (CK) level is not elevated in patients with a spell, this confirms PNES”. Similarly, in item 6, most participants (63%) responded that they would not induce a seizure.

**Figure 2:**
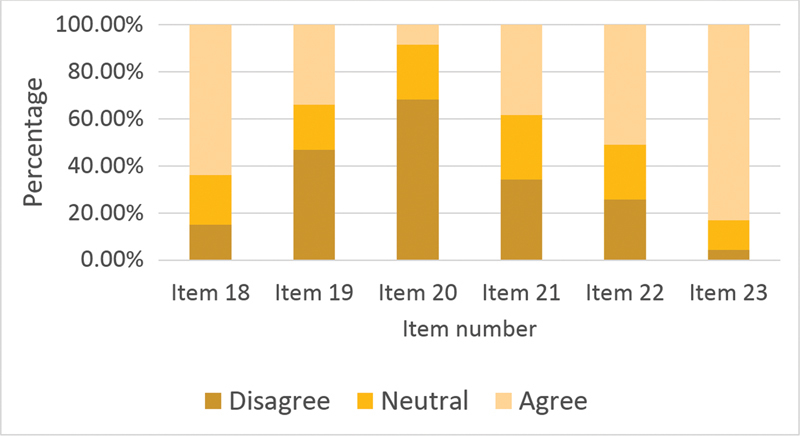
Percentage of agreement with items 18 to 23.

Figure 2 summarizes the results of items regarding the management of PNES patients. Question 23 was the item most agreed upon (83%): “It is difficult for PNES patients to accept the diagnosis”. Question 20 was the item most disagreed upon (68%): “Anti-seizure medications (ASMs) are the best treatment for PNES patients”.

**Figure 3:**
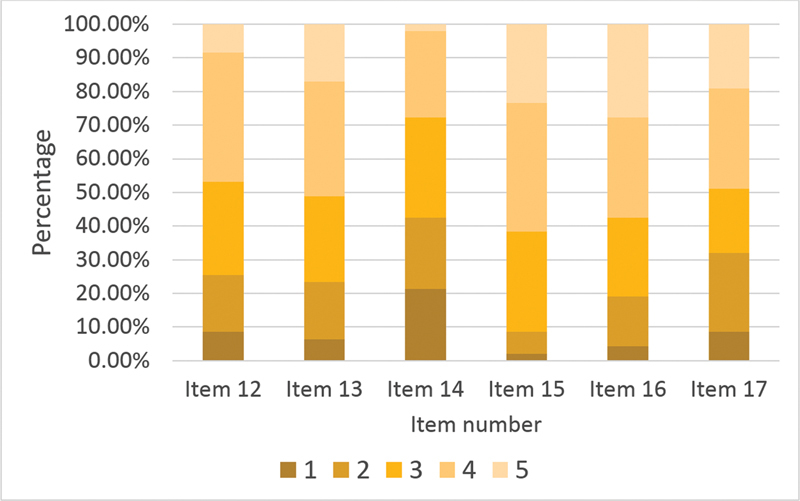
Percentage of agreement with items 12 to 17.

**Figure 4:**
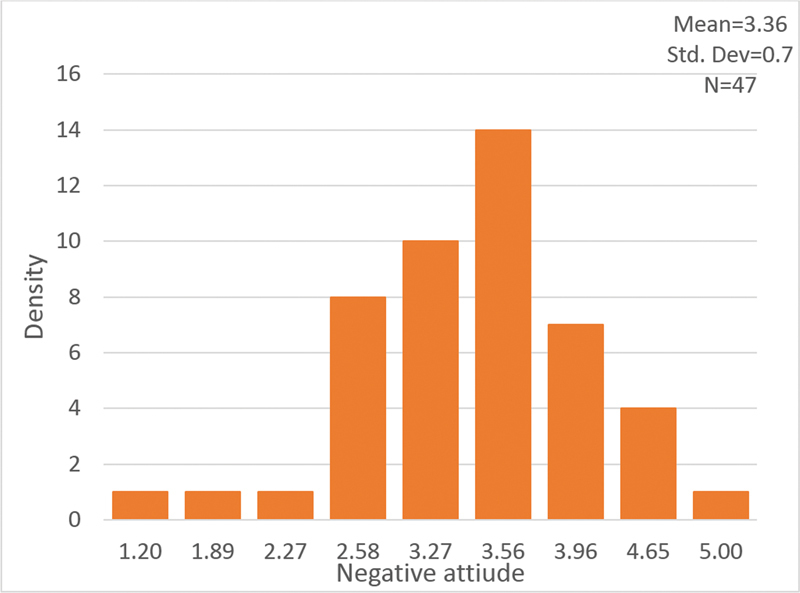
Histogram of the mean emotional attitude score towards PNES patients.

Items 12to 17 comprise the questions regarding emotional attitude toward PNES patients. Consistency measurements using the Cronbach test of all items showed α = 0.55 and 0.6 after removing item 14. The mean emotional attitude score of this section was of 3.3 ± 0.7 (range: 1.2 to 5). Figure 3 summarizes the percentage of agreement with emotional attitude statements, which comprised the emotional attitude variable. Question 15 was the item most agreed upon (61%): “PNES patients often arouse anger among medical staff”. Question 14 was the item most disagreed upon (42%): “PNES patients waste the time of medical staff”. Figure 4 summarizes the distribution of the mean emotional attitude score. Most of the responses scored above 3, meaning most participants had a negative emotional attitude towards PNES patients. The univariate analysis did not show a statistical difference in negative attitudes between genders, between nurses and physicians, or among different departments (neurology, internal wards, and ER). Furthermore, the interactions among the variables were statistically insignificant. We did not find a statistically significant correlation between negative attitudes and seniority (in years).

## DISCUSSION

We conducted the survey to check if what PNES patients feel regarding the harsh attitude of HCPs toward them is also reflected by the views of the HCPs.

The questionnaire also contained questions regarding knowledge, diagnosis, and management of PNES patients.

More than 80% of the participants agreed that PNES patients could have ES as well, and 65% agreed that lack of CK or prolactin elevation does not confirm PNES. Moreover, 65% agreed that they would not try to provoke a seizure, and almost 70% agreed that there is a need for long-term video-electroencephalographic monitoring (LTVEM) to confirm the diagnosis. Those results are better than the ones reported in previous surveys. 8 10

Furthermore, regarding the management of PNES patients, almost 70% of participants agreed that ASMs are not a good treatment for PNES, and 65% agreed that PNES patients should first be referred to a neurologist, reflecting the need for diagnosis confirmation before the psychological intervention. This finding is in alignment with those of previous surveys. 8 11

Overall, although there is still a need for improvement in terms of knowledge and management of PNES patients, this survey applied to a small sample highlighted that first responders from different disciplines at our center showed a high percentage of knowledge and good management practice of PNES patients.

On the other hand, most HCPs showed a negative emotional attitude towards PNES patients.

In total 62% of the participants agreed that PNES patients arouse anger among medical staff, while only 8% disagreed with this statement. Almost 60% of participants agreed that PNES patients are treated disrespectfully, and only 19% disagreed with this statement. Overall, 50% of the participants agreed that PNES patients require more attention than what is justified by their medical condition, they misuse medical resources, and are their cases are less urgent. Only 20% of the participants disagreed with those statements.

The only statement in this section that received a lower agreement score was question 14: “PNES patients waste the time of the medical staff”; 28% of the participants agreed, while 42% disagreed with this statement. The HCPs probably feel obligated to this group of patients like they would feel towards any other patients; hence, they do not regard them as time-wasters.

It is important to note that the questions regarding emotional attitude were formulated in the third person to assure honest responses from the participants.

Consistency measurements using the Cronbach test of all items regarding emotional attitude (questions 12 to 17) showed an α of 0.55 and 0.6 after removing item 14, which is relatively high for this survey applied to a small sample.

In general, studies that checked knowledge and attitudes among HCPs regarding patients with PNES 7 10 12 13 14 15 16 17 concluded that HCPs perceive PNES as hard to handle. In our survey, 83% of the participants thought that PNES patients have difficulty accepting the diagnosis. This result is higher than 73% of statement agreement in a previous survey. 10 This observation may reflect the difficulty of HCPs in communicating the diagnosis to PNES patients. 6 This difficulty may be caused by uncertainties HCPs have regarding the management of PNES patients.

These uncertainties are related to many factors:

- Uncertainty about the cause of PNES. Is it an organic or psychological disease?
PNES is defined by the International Classification of Diseases (ICD) as a dissociative phenomenon, while the Diagnostic and Statistical Manual (DSM) describes it as a conversion disorder. 2
- Uncertainty regarding PNES treatment: which discipline should treat those patients? What is the best way of treatment? In our survey, 70% of participants agreed that ASMs are not the treatment choice for PNES patients, but only 50% agreed they would discontinue ASMs in PNES patients.
- Epileptic seizures as a comorbidity of PNES. Between 10% and 50% of PNES patients are reported to have ES as a comorbidity. 18 In our survey, 83% of participants agreed with the statement regarding ES as a comorbidity. Hence, one can never be sure that a patient with PNES does not have comorbid epilepsy even after approval by LTVEM. Several studies have shown that many HCPs continue ASMs in patients with PNES because of this uncertainty, 19 20 although many clinical clues can differentiate ES from PNES in the same patient. 21

These uncertainties are even reflected by the name of the phenomena: PNES, which is defined by what it is not.

The uncertainty of HCPs regarding PNES leads to uncertainties among patients. As a result, HCPs may refer patients to do more ancillary tests, repeat LTVEM, and patients may repeatedly look for further consultations with different HCPs.

The uncertainties that HCPs and the patients experience can explain our results regarding the emotional attitude of HCPs toward PNES patients and are probably related to a feeling of helplessness of many HCPs regarding those patients. The difficulty explaining what the patients have can lead to feelings of anger and other negative feelings towards those patients.

The fact that we did not find any correlation regarding negative emotional attitudes and participants' department, occupation, or seniority implies that higher levels of knowledge and better management practices do not necessarily result in a more positive emotional attitude toward this group of patients.

Although teaching HCPs about PNES phenomena improves the differentiation of PNES and ES and the history-taking of those patients, 22 23 24 25 we have not found a study regarding the change in emotional attitude toward PNES patients through teaching. Our finding implies that the emotional attitude toward PNES patients is probably not solely based on cognition.

The present study suffers from several limitations.

- A small sample size.
- We did not use a control group of mental health personals.
- The study did not address possible reasons for negative emotional attitudes towards PNES patients.

On the other hand, the present single-center survey reflects the current situation of the management of ONES patients by the different disciplines that serve as first responders at our center. One of the survey's strengths is that 72% of the HCPs addressed filled out the questionnaire. This is relatively high compared to other surveys. 7 8 10

In conclusion, although the HCPs at our center have good professional practices regarding PNES patients, most reported a negative emotional attitude. This finding implies that what PNES patients feel regarding the harsh attitudes is also reflected by the views of the HCPs.

Emotional attitudes towards PNES patients may not depend solely on the level of knowledge of the HCPs.

Hence, there is a need for other educational methods such as group discussions or simulations focusing on the points of view of PNES patients. Furthermore, we believe that situation framing can have an impact on the patients' as well as the HCP's emotional attitudes. The transition from a view in which PNES patients do not have a “real disease”, consume resources, and their cases are less urgent to one in which PNES patients undergo real psychological distress, feel helpless, and can get better with appropriate professional help may help both the HCPs and the patients. These hypotheses need further research.
